# Early Brain Vulnerability in Wolfram Syndrome

**DOI:** 10.1371/journal.pone.0040604

**Published:** 2012-07-11

**Authors:** Tamara Hershey, Heather M. Lugar, Joshua S. Shimony, Jerrel Rutlin, Jonathan M. Koller, Dana C. Perantie, Alex R. Paciorkowski, Sarah A. Eisenstein, M. Alan Permutt, the Washington University Wolfram Study Group

**Affiliations:** 1 Department of Psychiatry, Washington University School of Medicine, St. Louis, Missouri, United States of America; 2 Department of Neurology, Washington University School of Medicine, St. Louis, Missouri, United States of America; 3 Department of Radiology, Washington University School of Medicine, St. Louis, Missouri, United States of America; 4 Department of Internal Medicine, Washington University School of Medicine, St. Louis, Missouri, United States of America; 5 Department of Neurology, University of Washington, St. Louis, Missouri, United States of America; Universidade Federal do Rio de Janeiro, Brazil

## Abstract

Wolfram Syndrome (WFS) is a rare autosomal recessive disease characterized by insulin-dependent diabetes mellitus, optic nerve atrophy, diabetes insipidus, deafness, and neurological dysfunction leading to death in mid-adulthood. WFS is caused by mutations in the *WFS1* gene, which lead to endoplasmic reticulum (ER) stress-mediated cell death. Case studies have found widespread brain atrophy in late stage WFS. However, it is not known when in the disease course these brain abnormalities arise, and whether there is differential vulnerability across brain regions and tissue classes. To address this limitation, we quantified regional brain abnormalities across multiple imaging modalities in a cohort of young patients in relatively early stages of WFS. Children and young adults with WFS were evaluated with neurological, cognitive and structural magnetic resonance imaging measures. Compared to normative data, the WFS group had intact cognition, significant anxiety and depression, and gait abnormalities. Compared to healthy and type 1 diabetic control groups, the WFS group had smaller intracranial volume and preferentially affected gray matter volume and white matter microstructural integrity in the brainstem, cerebellum and optic radiations. Abnormalities were detected in even the youngest patients with mildest symptoms, and some measures did not follow the typical age-dependent developmental trajectory. These results establish that WFS is associated with smaller intracranial volume with specific abnormalities in the brainstem and cerebellum, even at the earliest stage of clinical symptoms. This pattern of abnormalities suggests that WFS has a pronounced impact on early brain development in addition to later neurodegenerative effects, representing a significant new insight into the WFS disease process. Longitudinal studies will be critical for confirming and expanding our understanding of the impact of ER stress dysregulation on brain development.

## Introduction

Wolfram syndrome (WFS) is a rare (1 in ∼770,000) autosomal recessive genetic disease characterized by early childhood onset insulin dependent diabetes, optic nerve atrophy, vision and hearing loss, diabetes insipidus and neurodegeneration, resulting in death in middle adulthood, typically due to brainstem atrophy-induced respiratory failure [1]. There are no interventions to slow or stop this devastating deterioration. However, much is known about the mechanisms underlying these effects. The causative gene (*WFS1*) was identified by our group in 1998 [2], and a number of loss-of-function mutations have been described [2]–[4]. Cell [5] and animal models [6] have determined that *WFS1* encodes an endoplasmic reticulum (ER) membrane-embedded protein called wolframin [7], and that mutant forms of the WSF1 protein lead to disturbances of ER calcium homeostasis, driving ER stress-mediated apoptosis [8]–[10]. This process kills insulin producing pancreatic β-cells, leading to diabetes. WFS1 is also expressed throughout the brain, and cell death via ER stress is thought to underlie neurodegeneration in WFS [4], [11].

Although there are no current treatments for this disease, agents to treat ER stress-mediated apoptosis are currently in development [12], [13]. ER stress also has been implicated in a number of neurodegenerative (Alzheimer disease, Parkinson disease, amyotrophic lateral sclerosis), endocrine (Type 1 and Type 2 diabetes) and cardiovascular (heart disease, atherosclerosis) diseases [14]. Thus, WFS may provide a useful model for examining the impact of ER stress on the brain, particularly during neurodevelopment [15]. In contrast to the polygenic, multifactorial etiology of other diseases, WFS’s clinical features are due to deficiency of a single protein, wolframin. Research into this monogenic disease may provide critical insight into more genetically complex forms of diabetes and neurodegenerative diseases in which ER stress is implicated. Successful interventions for WFS may then provide the proof-of principle for using similar agents to treat more common and complex forms of ER stress-related diseases in the future. This innovative model has been followed successfully in other conditions (e.g. bone disorders [16]–[20]). An early step towards this important goal is to establish the characteristic neurodegenerative changes in WFS.

Our current knowledge of WFS-related brain changes come primarily from clinical exams and post-mortem neuropathological case studies of severely affected individuals with WFS. These studies found abnormalities in multiple regions of the brain, including hypothalamus, pituitary, pons, inferior olivary nucleus, lateral geniculate nucleus, thalamus, optic nerve, optic tract and cerebellum [1], [3], [21]–[31]. Motor, cognitive and psychiatric dysfunction have been reported in advanced WFS, but quantified, systematic assessments have not been performed in relatively early WFS. Clinical retrospective survey data suggest that neurological features occur late (15–30 yrs of age) in the disease process [32], [33], but these patients were not directly assessed with standardized, objective methodology or compared to age-equivalent controls [23], [30], [31]. Thus, it is not known at what stage of the disease these potential brain structural or functional changes or subsets of changes emerge, become clinically significant, or how structural and functional changes relate to each other and to overall disease severity.

An understanding of the natural history of neurological changes in WFS will be important in assessing the validity of animal models of WFS, for developing interventions for neurological changes, as well as addressing questions about how WFS1 mutations and ER stress affects neurodevelopmental processes. Thus, the goal of the current study is to determine the regional pattern of neuroanatomical abnormalities in WFS across a range of disease duration and imaging modalities. To address this gap in the literature, we studied individuals with WFS at the earliest stage of disease possible and compared them to both age and gender equivalent healthy and type 1 diabetic controls. Thus, we were able to survey the brain’s structural and functional integrity across a range of very early to more advanced disease using sophisticated *in vivo* structural brain imaging techniques and neurobehavioral measurements.

## Methods

Participants with WFS were evaluated clinically by a team of investigators including a pediatric neurologist, audiologist and ophthalmologist. Patients then performed neuropsychological testing and MRI scans and completed self-report questionnaires if over 16 (parents completed parent-report if 16 or younger) about psychiatric and behavioral issues. Control subjects completed MRI scans only.

### Subjects

#### WFS

Individuals with WFS who registered on the Washington University WFS Registry (http://wolframsyndrome.dom.wustl.edu/medical-research/Wolfram-Syndrome-Home.aspx), who were under the age of 30, aware of their diagnosis and able to travel were invited to participate. Further inclusion criteria were the diagnosis of diabetes mellitus and optic nerve atrophy before 18 years of age or genetic confirmation of a *WFS1* mutation.

#### Controls

MRI comparison groups consisted of age equivalent healthy controls (HC) and individuals with type 1 diabetes mellitus (Type 1 Controls or T1C) scanned at Washington University as part of other studies. Youth with T1C were recruited from the Pediatric Diabetes Clinic at St. Louis Children’s Hospital and Washington University School of Medicine in St. Louis. Non-diabetic controls were either healthy siblings of the diabetic patients or from the community. T1C were excluded for diagnosed psychiatric disorder, significant neurological history not due to diabetes, known premature birth (<36 weeks gestation) with complications, psychoactive medications, or physical limitations that would interfere with testing. No T1C participants had known retinopathy, nephropathy or neuropathy at the time of testing. Controls were excluded for current or past history of neurological and psychiatric diagnoses or other significant health conditions. All subjects underwent MRI scans for research purposes only, not for any clinical indication.

#### Ethics Statement

For all participants, informed consent was obtained prior to participation and the study was approved by the Human Research Protection Office at Washington University in St. Louis. Children under age 18 provided assent, and their parent/guardian provided written consent.

### Cognitive and Behavioral Testing

Before beginning testing, WFS participants were determined to have blood glucose levels above 70 mg/dl. Standardized IQ, cognitive and behavioral measures were administered. Each patient’s performance was compared to normative data for their age and gender.

*Vocabulary and Similarities* subtests from The Wechsler Abbreviated Scale of Intelligence (WASI) [34] were used to estimate a verbal intelligence quotient (IQ). The computerized *Conners’ Continuous Performance Test II (CPT II)* [35] was used to determine sustained attentional capacity. The *Letter Number Sequencing* [36], [37] task from the Wechsler Child or Adult Intelligence Scales was used to assess verbal working memory. The *Children’s or Adult California Verbal Learning Test (CVLT)* [38], [39] was used to assess verbal learning and memory. The *Achenbach Child Behavior Checklist or Adult Self Report* (CBCL, ages 6–16; ASR; ages 16 and above) [40] was used to assess parent and self-rated behavioral and mood status across the Internalizing Domain (Affective, Anxiety and Somatic Problems) and the Externalizing Domain (Attention Deficit/Hyperactivity, Oppositional Defiant and Conduct Problems).

### Neuroimaging

Before beginning scanning, WFS and T1C participants were determined to have blood glucose levels above 70 mg/dl. Scans were acquired on one of three Siemens 3T Tim Trio MRI scanners at Washington University. All three scanners had the same hardware and software, and they are each checked by Siemens maintenance personnel every 3 months to confirm that the machines are running in accordance with their recommendations. In addition, cross-calibration scanning on 15 normal control children from another study (unpublished data) using similar sequences as ours has shown a high degree of consistency and absolute difference in regional DTI FA (average r = .96, average mean difference in FA  = 2.3%) and Freesurfer-derived volumes (average r = .87, average mean difference in volumes  = 1.7%). These metrics are consistent with the literature of Freesurfer performance across different scanners [41], [42].

The following sequences were acquired: *T1-weighted MPRAGE sequence*: Sagittal acquisition, TR = 2400, TE = 3.16, TI = 1000, voxel resolution = 1×1×1mm, Time = 8:09 min. *T2-weighted MR:* Sagittal acquisition, TR = 3200, TE = 455, voxel resolution = 1×1×1mm, Time = 4:43 min. *T2 FLAIR:* Transverse acquisition, TR = 9190, TE = 98, TI = 2500, voxel resolution = .86×.86×3mm, Time = 3:59 min. This sequence was acquired for the WFS group only for clinical neuroradiological reading. *Diffusion Tensor Imaging (DTI):* The echo planar sequence consisted of 27 directions with the b-values ranging from 0 to 1400 s/mm^2^. Transverse acquisition, TR = 12300, TE = 108, voxel resolution = 1.98×1.98×2 mm, Time = 5:44 min. The DTI sequences in the control groups varied slightly in the number of b = 0 scans acquired, but their scans had the same resolution and range of b-values as the WFS group (with the exception of 16 subjects scanned with b-values only up to 1000 rather than 1400).

Methods were selected to provide complementary levels of information. The ROI method has several advantages over voxel-wise analyses – it minimizes the risk of type 1 error, avoids the many statistical assumptions of voxel-wise analyses, and allows for tailoring ROIs to avoid partial volume effects, thus reducing the impact of imperfect registration procedures [43]. On the other hand, voxel-wise or vertex-wise analyses consider all of the imaging space without regard for arbitrary or unreliable boundaries. We employ both approaches here for volumes and for diffusion tensor measures in order to provide a comprehensive view of the regional brain abnormalities in WFS. We pay particular attention to findings that are similar across imaging modalities and analysis methods.

### Regional Gray and White Matter Volumes

To determine regional gray and white matter brain volumes from anatomically defined regions, we used the semi-automatic segmentation program Freesurfer (v5.0) [44]. This program automatically reconstructed the brain from surface and volumetric registration to an atlas to quantify gray and white matter volumes. To minimize the opportunity for false positives, we focused on a select number of regions (n = 13: brainstem, cerebellar gray matter, cerebellar white matter, thalamus, pallidum, corpus callosum, hippocampus, amygdala, caudate, putamen, accumbens, total cortical gray, total cortical white matter). Left and right volumes were averaged and corrected for intracranial volume (ICV or volume within the skull, derived from Freesurfer) according to validated methods [45]. A Bonferonni multiple comparison correction procedure was applied to determine significance, after controlling for age and gender.

### Brainstem Segmentation Volumes

To examine the brainstem, known to be particularly affected in advanced WFS, in more detail, we performed manual segmentation of the Freesurfer-generated brainstem according to published criteria [46]. The brainstem was divided into midbrain, pons and medulla using anatomical landmarks. The border between the midbrain and the pons was defined by a plane touching both the upper rim of the pons and the inferior border of the inferior colliculus. The border between the pons and medulla was defined by a plane parallel to that of the midbrain-pons border, touching the lower rim of the pons. The MR images were rotated to align the brainstem so that these borders were transverse slices, and the Freesurfer-generated brainstem was segmented according to those slices. Two raters independently chose these landmarks (intraclass correlation coeffecients [ICCs] were above .98 for all landmarks) and the chosen rotations were averaged. Analyses were normalized using ICV and controlled for age and gender.

### Surface-based Cortical Measurements

To explore cortical metrics in a landmark-independent manner, we reconstructed and segmented individual subjects’ cortical surfaces using Freesurfer (v5.0) [47]–[49]. The cortical gray/white matter border and pial surfaces were identified in each subject, and a triangular tessellation was applied across the cortical surface. Using these cortical maps, we calculated multiple surface-based measurements at each vertex of the triangular mesh. These included cortical thickness (the distance between the white and pial surfaces), surface area (the sum of the areas of the triangles connected to a vertex) and gray matter volume (the product of cortical thickness and surface area). A whole brain, vertex-by-vertex group comparison was conducted using Freesurfer’s group analysis tool, Qdec. Effects between our patient group and our controls (healthy and T1DM controls collapsed due to restrictions in the statistical modeling of this software) were calculated for each hemisphere by general linear model (GLM) at each vertex for thickness, area, and volume, while controlling for age and gender (thickness), or age, gender, and ICV (for area and volume). Data were smoothed using a full width/half-maximum Gaussian kernel of 15 mm and corrected for multiple comparisons using Monte Carlo permutation cluster analyses with a significance threshold of p<.05.

### Whole Brain Voxel-wise Analyses of Gray and White Matter Volumes

Voxel-based morphometry (VBM) was performed with statistical parametric mapping software (SPM8; Wellcome Department of Cognitive Neurology, www.fil.ion.ucl.ac.uk). Images were simultaneously normalized to Montreal Neurological Institute (MNI) space using non-linear transforms, corrected for intensity inhomogeneity, and segmented into gray matter, white matter, and CSF. We used custom tissue probability maps based on our subjects instead of the default SPM maps to improve segmentation [50], [51]. Gray and white segments were modulated to produce images representing gray and white matter volume [50]. After this processing, voxel dimensions were 2×2×2 mm. Modulated segments were smoothed with a 12 mm full width half maximum Gaussian kernel to promote normality of residuals [52]. Voxels with segmented intensities less than 0.1 were masked out with an absolute threshold to reduce voxels possibly belonging to other tissue classes and since these voxels are less likely to adhere to assumptions of normality [53]. Images were analyzed by SPM8, comparing groups (WFS vs. all controls) at each voxel, which results in statistical parametric maps (SPMs) on which every voxel’s intensity corresponds to a t value. The SPMs were then thresholded to show only voxels with t values corresponding to uncorrected p<.001. The probability of resulting clusters was corrected for multiple comparisons taking into account non-uniformity due to intrinsically inhomogeneous smoothness using the stat_threshold script from Worsley’s fmristat package [54], [55]. Age and gender were removed as covariates from all models. Independent sample t-tests were performed for comparisons between groups, defining contrasts in each direction (e.g., WFS > controls; WFS < controls). Cluster-level multiple comparison and smoothness corrected p values <.05 were considered significant.

### General Analysis Approach to White Matter Microstructural Integrity

DTI images were atlas-transformed and measures of white matter microstructural integrity (fractional anisotropy or FA and mean diffusivity or MD) were computed [56], [57]. These measures are sensitive indicators of white matter injury [58] and can detect changes even when standard T2-weighted images appear normal and the volumes of white matter regions are similar [59]–[63]. We computed FA and MD volumes from native space data and analyzed these images in two complementary ways, to balance the desire to control type 1 error while fully exploring our unique dataset.

### Regions of Interest DTI Analyses

Regions were selected based on a well-established DTI atlas [64] and were checked by a neuroradiologist with expertise in DTI. We then compared the placement of the regions on each subject’s colorized FA map, T1 and T2 images simultaneously. ROIs were shifted by a few voxels as necessary to better conform to each individual’s native anatomy. Finalized regions were then applied to FA and MD volumes. Raters have established ICC’s above .90 for FA and MD values for all ROIs. To minimize the opportunity for false positives, we focused on a select number of regions (n = 10: cerebellum, optic radiation, posterior limb of the internal capsule, corpus callosum [average of 3 regions], centrum semiovale, thalamus, putamen, hippocampus, cerebellar peduncle, and pons), average left and right volumes, applied Bonferroni multiple comparison correction (.05/10 or p<.005), and controlled for age and gender.

### Tract-Based Spatial Statistics (TBSS) DTI Analyses [65]

Despite the advantages of the ROI method of DTI, we also used voxel-wise exploratory analyses of our DTI data to confirm our ROI findings but also to be open to white matter effects in other locations without regard for anatomical boundaries. Diffusion weighted images were internally motion corrected using 9-parameter affine (rigid body + scanner axis stretch) registration using in-house developed software. Then, using each individual’s motion-corrected, aligned, and averaged DWI data set, BET (FMRIB Brain Extraction Tool) was used to compute a brain mask, and FDT (FMRIB diffusion toolbox) was used to compute FA maps. To create a target template for registration, a subset of individuals were chosen from the entire subject pool to provide an even distribution of males and females and ages. FA images in the target group were then nonlinearly aligned to 1×1×1 mm space and to each other. The target image was the image that requires the least amount of warping for all other FA images to align to it. All FA images in the entire subject pool were then nonlinearly aligned to each other in 1×1×1 mm space and to the target image. The target image was affine-aligned into MNI152 standard space, and every FA image was then transformed into 1×1×1 mm MNI152 space. A mean FA image was calculated and used to produce the mean FA skeleton to represent the center of white matter tracts. FA images were projected onto the mean FA skeleton and thresholded at FA = 0.2 for voxel-wise analyses. Mean, axial and radial diffusivity images were also calculated and all were analyzed with Randomise, a permutation-based multiple comparisons corrected statistical approach [66]. Age and gender were co-varied in analyses comparing groups (WFS vs all controls).

### Statistical Analyses of Neuroimaging Data

For all region-based analyses we performed univariate analyses of each region, with group as the independent variable (HC, T1C, WFS) with age and gender covaried. We were then able to determine if control groups differed on regional values. We applied a Bonferroni correction for multiple comparisons within imaging modalities. For gray and white matter volumes of interest, the criterion for significance was p<.0038 (.05/13). For DTI regions of interest, the criterion for significance was p<.005 (.05/10). Regions that survived correction from any analyses were examined for differences between groups with post-hoc comparisons. For voxel or vertex-wise analyses, we compared all WFS vs all controls, to simplify the statistical models needed (and since no regional differences were found between control groups) and to maximize power. Each analysis used software-specific multiple comparison correction procedures described above, and age and gender were removed as covariates from all models.

## Results

### General Clinical Information for WFS Group

Patients with WFS ranged in age from 5.9 to 25.8 (mean = 14.6, SD = 6.1; 6 male 8 female). All had confirmed mutations on the *WFS1* gene and had insulin dependent diabetes mellitus (average age of onset = 5.8 years; range 2–14 years of age). All but one had been diagnosed with optic nerve atrophy at the time of assessment (average age at diagnosis = 9.3 years; range 3 to 16 years of age); 10/14 were being treated for diabetes insipidus (average age of diagnosis = 10.1 years; range 2.5 to 17 years of age). WFS individuals had a wide range of duration of diabetes mellitus (range 0.75–21.7 years, mean = 8.8, SD = 6.0), optic atrophy (range 1.1–15.2 years; mean = 5.9, SD = 4.9) and diabetes insipidus (range .25 to 11.9 years; mean = 5.2, SD = 4.5). Younger individuals tended to have shorter duration of all of these symptoms. Standard ophthalmological tests revealed that 13/14 WFS participants had color vision impairment ranging from mild to severe. All had abnormally thin retinal nerve fiber layers on optical coherence tomography (OCT). Audiometric tests revealed that 7/14 patients had evidence of hearing loss. Clinical neurologic exams revealed that the most common abnormalities were horizontal nystagmus and difficulty tandem walking (8/14 subjects). Several subjects (5/14) had impaired vibration sense, and other more subtle motor abnormalities were also noted, including increased lower extremity tone, intention tremor, and brisk deep tendon reflexes in one subject each. These clinical findings confirm that our WFS group ranged from very mild to moderate severity of symptoms on standard clinical measures.

### Cognitive/Behavioral Information

Some individuals with WFS, either due to language (non-native English speaker, n = 1) or sensory issues (significant vision problems, n = 2), could not complete all of the tasks. However, the rest of the WFS group performed above the average range for verbal intelligence (Verbal IQ mean percentile  = 77, SD = 15), and within the average range for verbal learning and memory (CVLT Total Recall, trials 1–5, mean percentile  = 57, SD = 25; CVLT Long Delay Free Recall mean percentile  = 55, SD = 25), and attention (CPT Detectability mean percentile  = 41, SD = 27). In contrast, on a comprehensive psychological questionnaire, participants or parents reported more symptoms in the Internalizing Domain which includes depression, anxiety and somatic symptoms (mean percentile for symptoms = 65.6, SD = 32) than in the Externalizing Domain which includes conduct disorders, impulsivity and aggression (mean percentile for symptoms = 33.1, SD = 24; paired t-test, t(13) = 3.85, p = .002). Five individuals had clinically significant symptoms (T score above 69) and 1 had clinically borderline symptoms (T score 65–69) in the Internalizing domain, but only 1 had clinically significant symptoms in the Externalizing Domain. Eleven WFS subjects had blood glucose levels recorded at the time of testing; levels ranged from 124 to 325 (mean = 225 mg/dl, SD = 65).

### Neuroimaging

MRIs were acquired on 11 individuals with WFS. The remaining 3 patients had contraindications for MR and could not be safely scanned (cochlear implant, n = 2; bladder neurostimulator, n = 1). Two of the 11 had clinically normal scans; the other 9 had clinically detectable abnormalities on MR similar to those noted in case studies of WFS (i.e. 5 had elevated T2 signal in pons; 7 had elevated T2 signal in frontal and occipital cortex) and individuals with diabetes insipidus (i.e. 5 had absent or reduced normal pituitary bright spot on T1).

De-identified scans from age and gender-equivalent HC and T1C individuals were obtained for comparisons to the WFS group. These subjects either had T1-weighted images (for gray and white matter volume measures; HC, n = 30; T1C, n = 22) or DTI scans (HC, n = 45; T1C controls, n = 10). Thirty-eight subjects had T1-weighted scans and 12 had DTI scans on the first scanner, 25 (including the 11 WFS subjects) had T1-weighted scans and 38 (including the 11 WFS) had DTI scans on the second scanner and no subjects had T1-weighted scans, and 16 had DTI scans on the third scanner. Within DTI sequences, 50 subjects had b1400 and 16 had b1000 scans; 42 had 25 directions and 24 had 26 direction scans. The control groups were not different in mean age or gender distribution from the WFS group (Table 1). In both T1C and WFS groups, duration of diabetes was highly correlated with age (r>.80). The WFS group and the larger T1C group (n = 23 with T1-weighted MR) were not different in mean duration of diabetes. However, the smaller T1C group (n = 10 with DTI) had significantly shorter duration of diabetes than the WFS group (Table 2). For T1C and WFS subjects, glucose values were measured just before or just after scans, and ranged from 71–337 for the T1C group (n = 22) and 123–289 for the WFS group (n = 8). Mean values were not different between groups (t-test, t = -.74, p = .47; T1C mean = 182 mg/dl, SD = 70; WFS mean = 203 mg/dl, SD = 62).

**Table 1:** Mean (±SD) age and gender distribution and clinical variables for control groups and WFS group for MPRAGE scans.

		HC (n = 30)	T1C (n = 22)	WFS (n = 11)
**Gender**	M/F	15/15	14/8	5/6
**Age in years**	Mean	12.5±3.2	14.3±3.2	14.0±6.3
	Range	7.6–18.7	7.1–19.0	5.9–25.8
**Duration of diabetes in years**	Mean	–	4.5±4.7	7.4±5.3
	Range	–	.11–13.6	.75–16.2

Abbreviation. HC: healthy controls; T1C: diabetic controls; WFS: Wolfram Syndrome group; SD: standard deviation.

There were no differences between groups for age (p = .23), gender (p = .51) or duration of diabetes between T1C and WFS (p = .12).

**Table 2:** Mean (±SD) age and gender distribution and clinical variables for control groups and WFS group for DTI scans.

		HC (n = 45)	T1C(n = 9)	WFS (n = 11)
**Gender**	M/F	23/22	7/2	5/6
**Age in years**	Mean	11.9±3.1	11.8±2.3	14.0±6.3
	Range	7.0–17.7	7.1–14.2	5.9–25.8
**Duration of diabetes in years**	Mean	–	.19±.05	7.4±5.3
	Range	–	.11–.25	.75–16.2

Abbreviation. HC: healthy controls; T1C: diabetic controls; WFS: Wolfram Syndrome group; SD: standard deviation.

There were no differences between groups for age (p = .24) or gender (p = .28) but there was a significant. difference in duration of diabetes between T1C and WFS (p = .001).

### Regional Gray and White Matter Volumes

Overall, there was a significant effect of group (WFS, HC, T1C) on intracranial volume (ICV) after correcting for age and gender (F(2,58) = 5.9, p = .005). Post-hoc pair-wise comparisons revealed that the WFS group had smaller ICV compared to the HC and T1C groups (p<.004), but these control groups were not different from each other (p = .91). Whole brain volume (defined as everything inside the pial surface of the brain, excluding brainstem and cerebellum) was also smaller in the WFS group (F(2,58) = 5.1, p = .009), but after controlling for ICV, was similar across groups (F(2,58) = 0.9, p = .38).

**Figure 1:**
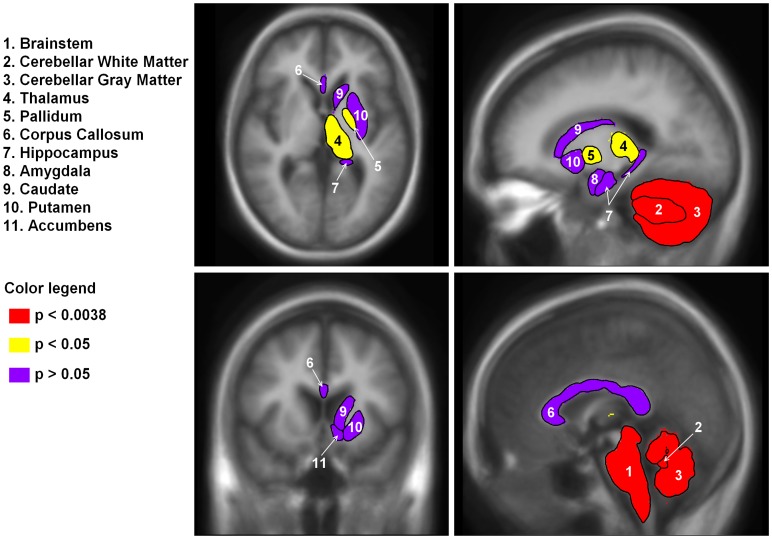
Regional subcortical brain volumes by significance level. Regions were segmented in Freesurfer. Left and right volumes were averaged and are shown on the right side of the brain only. The brainstem, cerebellar gray matter and cerebellar white matter were significantly reduced in the WFS compared to controls and survived Bonferroni multiple comparison correction (in red; p<.0038). In addition, the thalamus and pallidum were also reduced in WFS compared to controls, but did not survive correction (p<.05, in yellow). Finally, the corpus callosum, hippocampus, amygdala, caudate, putamen and accumbens were not different between groups (p>.05, in purple).

Out of 13 regions normalized for ICV, 5 had a main effect of group at the p<.05 level; 3 of these survived multiple comparison correction (p<.0038)(Table 3; Fig 1). These regions were brainstem (F(2,58) = 22.5, p<.001), cerebellar gray matter (F(2,58) = 8.3, p = .001) and cerebellar white matter (F(2,58) = 14.7, p<.001). In all of these regions, the WFS group had smaller volumes than both the HC and T1C (ps<.05), but the HC and T1C groups did not differ from each other (p>.05). In addition, even the youngest and least affected WFS patients showed reduced volumes in these regions compared to control groups.

**Table 3:** Mean (± SEM) volumes of brain regions in mm^3^, adjusted for age and gender.

Brain Region (mm^3^)	HC (n = 30)	T1C (n = 22)	WFS (n = 11)	p value
Intracranial (ICV)	1481637±134316	1509633±127999	1358061±92086 a,b	.005
Whole brain	1029284±94162	1045945±89363	940183±84097 a,b	.009
Total cortical gray	538,744±4231	535030±4952	538,660±6919	.84
Total cortical white	443,439±4787	456,997±5603	447,066±7829	.19
Brainstem	20,560±343	19,960±401	16,220±560 a,b	**<.001**
Cerebellum white	15,635±326	14,772±382	12,236±534 a,b	**<.001**
Cerebellum gray	58,419±761	58,314±891	52,771±1245 a,b	**.001**
Thalamus	7,168±92	7,112±107	6,585±150 a,b	.005
Pallidum	1,775±29	1,753±34	1,630±48 a,b	.04
Total corpus callosum	3,016±70	3,138±82	2,883±115	.19
Hippocampus	4,306±58	4,180±68	4,083±95	.12
Amygdala	1,706±26	1,716±31	1,650±43	.44
Caudate	3,771±89	3,868±105	3,987±146	.44
Putamen	6,145±106	6,120±125	6,182±174	.96
Accumbens	696±19	699±23	756±32	.24

Abbreviation. HC: healthy controls; T1C: diabetic controls; WFS: Wolfram Syndrome group; SEM: standard error of the mean.

P values shown are for the main effect of group in univariate GLM analyses for each measure.

Values in **bold** survived Bonferroni correction for multiple comparisons (p<.0038).

a different from HC group; ^b^different from T1C group.

### Brainstem Segmentation Volumes

**Figure 2:**
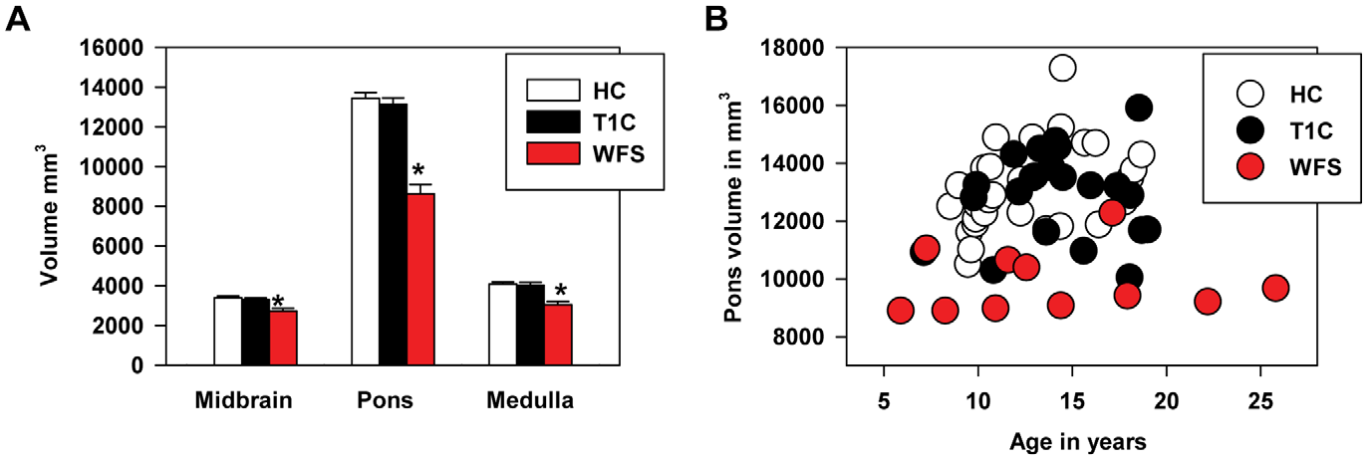
Volume of brainstem segments by diagnosis and age. (A) The WFS group has reduced volumes in all three brainstem segments after adjusting for intracranial volume. A repeated measures general linear model analysis found a segmental volume by diagnosis interaction (F(4,112) = 7.6, p<.001). Volume in the WFS group was significantly different from the control groups for all three segments (*), but the effect was most striking in the pons. (B) The pons appeared to be reduced in volume in almost all WFS individuals even after adjusting for intracranial volume. This figure shows the relationship between pontine volume and age. HC  =  healthy controls; T1C  =  diabetic controls; WFS  =  Wolfram group.

Volumes of brainstem segments (midbrain, pons, medulla) were normalized for ICV. A repeated measures general linear model analysis found, as expected, a main effect of diagnosis (F(2,56) = 22.4, p<.001; WFS < T1C and HC) and a main effect of segment (F(2,55) = 41.3, p<.001; pons > midbrain and medulla) with age and gender as covariates. More interestingly, we found a segmental volume by diagnosis interaction (F(4,112) = 7.6, p<.001) that was still significant after also covarying duration of diabetes (WFS vs T1C only, F(2,26) = 9.1, p<.001). Post-hoc contrasts found that the WFS group volume was significantly different from the control groups for all three segments, but the effect was most striking in the pons (Fig 2A). Before normalization, 10/11 WFS patients had reduced volume in the pons compared to age equivalent controls. After normalization, 8/11 WFS patients had reduced volume in the pons compared to age equivalent controls (Fig 2B).

### Surface-based Cortical Measurements

**Figure 3:**
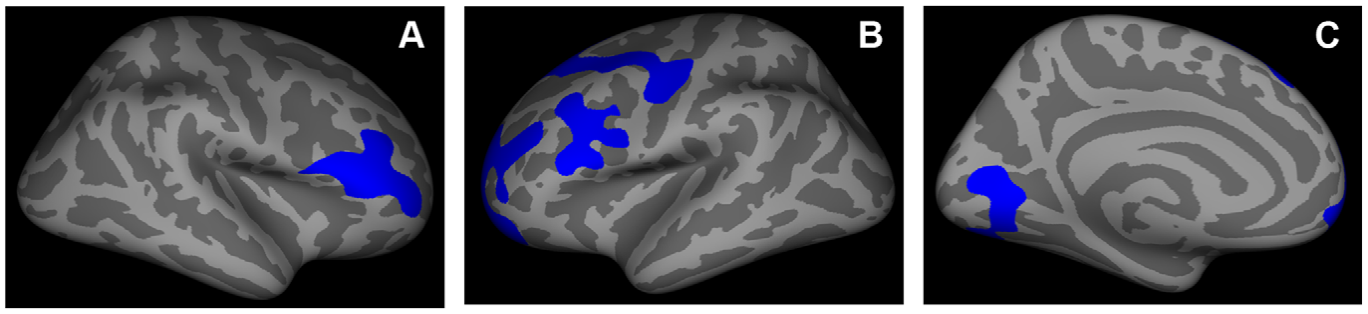
Regions with cortical thinning in the WFS group. After multiple comparison correction and adjustment for age and gender, (A and B) the rostral middle frontal cortex was found to be thinner bilaterally (right cluster-wise p = .001; left cluster-wise p = .0001 and p = .0304), as was (B) the left precentral (cluster-wise p = .0008) and (C) the left lingual (cluster-wise p = .0113) cortex.

We examined cortical thickness, cortical area and cortical volume (a product of thickness and area) between the combined control group and the WFS group across the entire cortex. In vertex-wise analyses, the WFS group had reduced thickness in several regions after controlling for age and gender, and applying multiple comparison corrections (Fig 3). In the left hemisphere, WFS had reduced thickness in a precentral region (BA 6; cluster size  = 2311.73 mm^2^, cluster-wise p  = .0008), a lingual region (BA 18; cluster size  = 1676.56 mm^2^, cluster-wise p  = .0113), and two rostral middle frontal regions (BA 10; cluster-size  = 3060.86 mm^2^, cluster-wise p  = .0001; BA 9; cluster size  = 1422.12 mm^2^, cluster-wise p  = .0304). In the right hemisphere, there was also reduced thickness in the WFS group in a rostral middle frontal region (BA 46; cluster size  = 2320.65 mm^2^, cluster-wise p  = .001). No clusters from the surface analyses of surface area or volume had significant group effects after multiple comparison correction.

### Whole Brain Voxel-wise Analyses of Gray and White Matter Volumes

**Figure 4:**
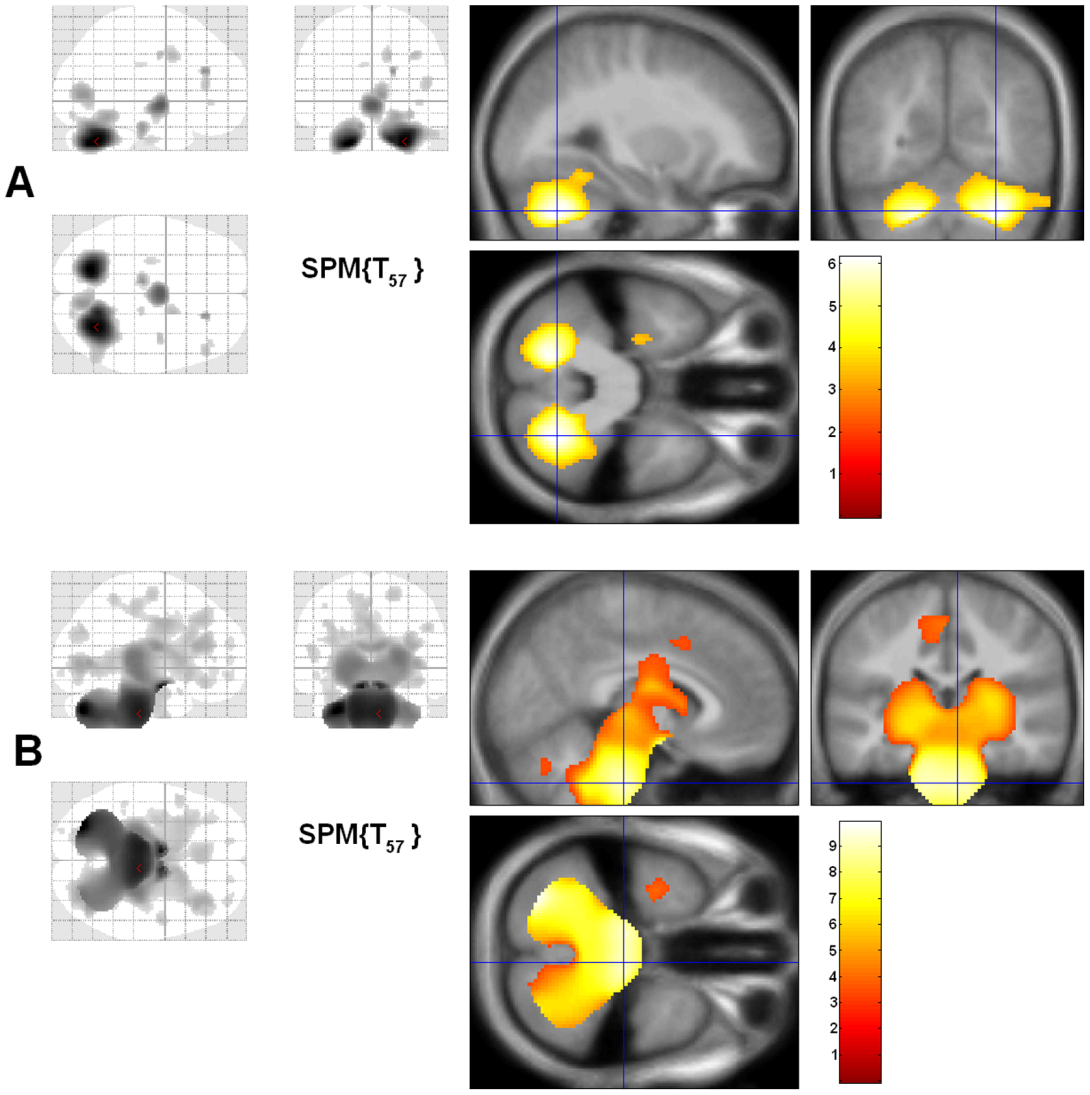
Voxel-based morphometry (VBM) findings. Gray and white matter clusters where WFS have lower volumes than controls. (A) Gray matter clusters included right cerebellum (p = .0008), and left cerebellum (p = .0125), while (B) white matter clusters included a large cluster consisting of much of the cerebellum, brainstem, and subcortex (p<.001), and a small cluster in the parietal-occiptal cortex (p = .0239). Glass brain (all results shown collapsed on a single slice) view shown on left, and the significant cluster on an average MR overlay is shown on the right. Cross hairs are placed in the voxel with a peak t value in the cluster.

VBM analyses of gray matter revealed that the WFS had significantly less gray matter volume in the cerebellum. Two large clusters survived multiple comparison and smoothness correction, covarying for age and gender: one in the right cerebellum (p = 0.0008, 3278 voxels) and one in the left cerebellum (p = .0125, 1740 voxels)(Fig 4A). There were no clusters in which WFS had more gray matter volume than controls. VBM analyses of white matter revealed that the WFS group had significantly lower volumes in one very large cluster that included much of the cerebellum bilaterally and extended through the brainstem and subcortex (p<.001, 29678 voxels)(Fig 4B). An additional, much smaller cluster was found in the parietal-occipital cortex (p = .0239, 243 voxels). There were no significant clusters in which the WFS group had more white matter volume than controls.

### Regions of Interest DTI Analyses

Placed regions were visually verified to not overlap with any of the T2 signal abnormalities observed in WFS patients. Out of the 10 regions examined for DTI measures, 3 had a main effect of group for FA and 3 had a main effect of group for MD measures at the p<.05 level. Two FA regions (cerebellum: F(2,60) = 13.7, p<.001; optic radiation: F(2,60) = 11.0, p<.001) and one MD region (cerebellum: F(2,59) = 14.0, p<.001) survived multiple comparison correction with age and gender as covariates (main effect of group, p<.005) (Table 4). For FA in the optic radiations, the WFS group was significantly different from the HC group (p<.05), but not the T1C group (p = .18), and the two control groups were different from each other (p<.05). For FA in the cerebellum, the WFS group was significantly different from both control groups (p<.003), but the control groups were not different from each other (p = .37). For MD in the cerebellum, all three groups were significantly different from each other (p<.001; post hoc comparisons).

**Table 4:** Mean (±SEM) DTI measures from selected brain regions and adjusted for age and gender.

BrainRegion	Measure	HC(n = 45)	T1C(n = 9)	WFS(n = 11)	p value
Cerebellum	FA	.23±.004	.22±.01	.18±**.**01 a,b	**<.001**
	MD	.70±.01	.66±.02	.77±.01 a,b	**<.001**
OpticRadiation	FA	.55±.01	.50±.02 a	.47±.02 a	**<.001**
	MD	.84±.01	.81±.02	.87±.02	.20
Corpuscallosum	FA	.74±.01	.77±.01	.71±.01 ^b^	.009
	MD	.80±.01	.72±.03	.80±02 ^b^	.03
PLIC	FA	.68±.01	.69±.01	.68±.01	.68
	MD	.69±.01	.66±.01 a	.68±.01	.04
Centrumsemiovale	FA	.33±.01	.38±.02	.35±.02	.12
	MD	.84±.02	.75±.05	.76±.05	.11
Thalamus	FA	.31±.01	.30±.02	.30±.02	.47
	MD	.75±.01	.73±.02	.73±.01	.19
Putamen	FA	.13±.004	.13±.01	.14±.01	.86
	MD	.71±.004	.69±.01	.69±.01	.06
Hippocampus	FA	.17±.01	.16±.01	.14±.01	.10
	MD	.88±.03	.85±.06	.97±.05	.22
Cerebellarpeduncle	FA	.56±.02	.60±.03	.57±.03	.57
	MD	.67±.01	.63±.02	.66±.02	.08
Pons	FA	.45±.01	.42±.02	.40±.02 a	.03
	MD	.72±.01	.74±.02	.71±.02	.65

Abbreviation. HC: healthy controls; T1C: diabetic controls; WFS: Wolfram Syndrome group; SEM: standard error of the mean; PLIC: posterior limb of the internal capsule.

P values shown are for the main effect of group in univariate GLM analyses for each measure.

Values in **bold** survived Bonferroni correction (p<.005) for multiple comparisons.

a different from HC group; ^b^different from T1C group.

### Tract-Based Spatial Statistics (TBSS) DTI Analyses

**Figure 5:**
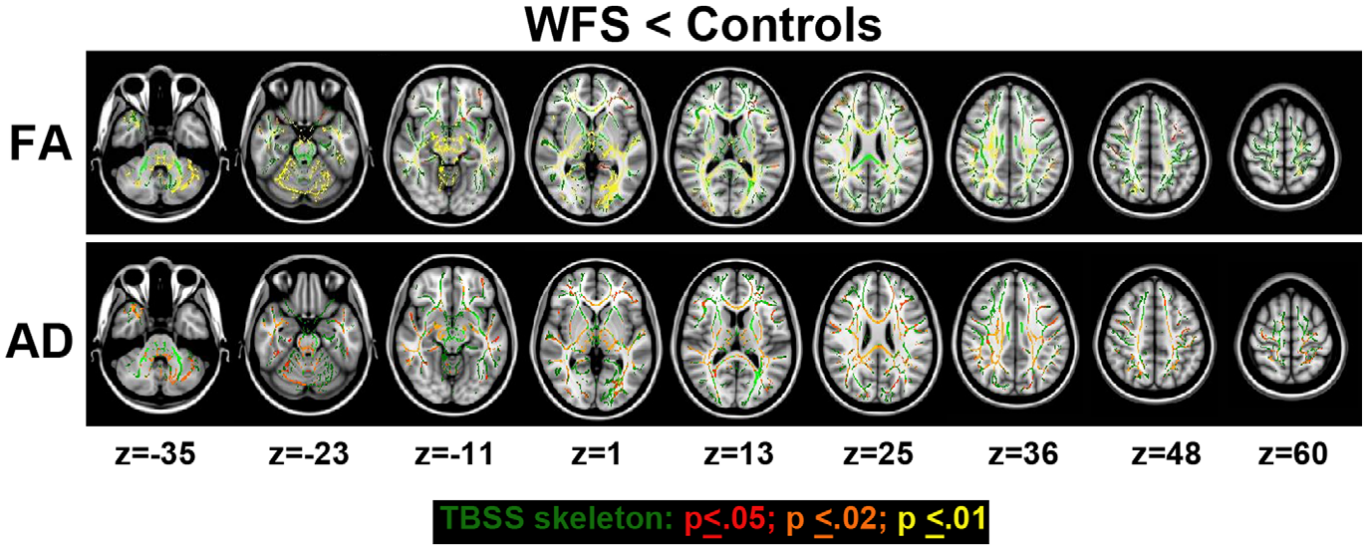
Results from tract-based spatial statistics (TBSS) analysis. Fractional anisotropy (FA) and axial diffusivity (AD), but not radial diffusivity, were reduced in the WFS group compared to controls (healthy and diabetic control groups combined), even after multiple comparison correction. There were no significant findings in the opposite direction (WFS > controls). White matter tracts in the cerebellum, brainstem, and optic radiations were prominently affected, but changes were also noted in other areas as well. The multiple comparison corrected p values are represented in the color coding. Z  =  the Talairach coordinate for the transverse plane.

Significant reduction in FA was found in the WFS group across a wide range of regions after controlling for age and gender and correcting for multiple comparisons. The largest and most striking clusters were found in the brainstem, cerebellum and optic radiations (see Fig 4), consistent with the results from ROI analyses. In addition, we detected widespread decreased FA in the subcortical white matter, which is not thoroughly sampled by ROIs. The WFS had reduced axial, but not radial, diffusivity in the cerebellum and brainstem primarily, although differences were seen across the brain to a lesser extent, similar to the FA results (Fig 5).

### Scanner and Sequence Differences in Neuroimaging Data

To assess any impact of differences in scanners, we performed further analyses on the significant regions, restricting analyses to only those subjects with data collected on the same 3T scanner. For subcortical volumes (HC, n = 4, T1C, n = 10, WFS, n = 11) and DTI measures (HC, n = 17, T1C, n = 9, WFS, n = 11) analyses revealed the same pattern as our larger sample (main effect of group; p<.001 for brainstem, cerebellar white, cerebellar gray, cerebellar FA and MD, and optic radiation FA; group x segment interaction for brainstem, p<.001). These results survive multiple comparison correction and support the robustness of our findings, suggesting that scanner and sequence variability within our control groups did not bias the original results.

## Discussion

We report quantitatively measured and regionally distinct structural brain changes in early stage WFS. We found reduced ICV, dramatically reduced regional volumes and altered white matter microstructural integrity. The brainstem, cerebellum and optic radiations were particularly affected in WFS compared to both healthy controls and controls with type 1 diabetes (T1C). Notably, decreased brainstem and cerebellum volumes (after controlling for intracranial volume or ICV) were seen in almost all individuals with WFS, regardless of age or duration of diabetes or other features of the disease. These data together suggest that WFS affects brain development both globally and preferentially, even at the earliest stages of symptoms. Previous clinical retrospective survey studies suggested that neurological features occur later (15–30 yrs of age) in the disease process [32], [33], which led to the assumption that brain changes in WFS also occur later and are neurodegenerative in nature. Our findings, based on direct, objective and quantified measures of brain structure and function, counter this assumption by showing abnormalities at the onset of classic WFS symptoms (diabetes and optic nerve atrophy), are consistent with abnormal neurodevelopment, and so represent a significant new insight into the WFS disease process.

Our observation that individuals with WFS have significantly reduced ICV, in comparison to both diabetics and healthy controls, supports the concept of aberrant neurodevelopment in WFS. The major expansion of the cranial vault is driven by brain growth early in childhood until all of the cranial sutures fuse (around age 6), at which point ICV changes only very slowly [67], [68]. Skull maturation is 77% complete by age 2 [69], 90% complete by age 6 [68], and maximum growth is achieved at about 10–13 years of age [70]. ICV is therefore considered to be a reflection of how fully the brain grew and matured in childhood [71]. For example, neurodevelopmental studies of autism [72], Rett Syndrome [73], and childhood onset multiple sclerosis [74] have interpreted differences in ICV or head circumference between patient and control groups as an indication of abnormally accelerated or decelerated brain growth during development. In both autism and Rett Syndrome, individuals fall within the normal range at birth, but within the first two years of development their brain growth rapidly diverges from the normal trajectory, and the degree of divergence correlates with the core symptoms of the disorders [72], [73]. Based on the literature, we suspect that the reductions in ICV between our WFS group and both the diabetic and healthy control groups may indicate that WFS affects the brain early in the course of development. We did not see differences in whole brain volume once we corrected for reduced ICV, which is evidence against global brain degeneration.

The preferential vulnerability of the brainstem and cerebellum in WFS has been described previously, generally in adults in advanced disease states, without quantification or control groups [1], [3], [21]–[31], [75], [76]. These qualitative findings typically have been interpreted as reflecting a neurodegenerative rather than a neurodevelopmental process [21]. Our study quantifies this preferential vulnerability across multiple imaging modalities and analysis approaches (VBM, Freesurfer regions, DTI regions of interest, TBSS) and at a very young age. Our data clearly indicate that brainstem and cerebellum volume abnormalities are present at the time of the very earliest clinical manifestations of WFS (e.g. 0.5 years of diabetes). In combination with the ICV results, this novel observation suggests that the brain may be affected in WFS from a very early stage. Interestingly, the cerebellum and the brainstem follow a very early, prolonged and neurobiologically-linked course of development in humans [77], which is thought to make them more susceptible to neurodevelopmental disorders [78]. Longitudinal follow-up of our cohort will be critical in determining if individuals show further changes within these structures relative to normal developmental trajectories and at what stage of disease severity.

In addition to these profound subcortical and cerebellar changes, we also found relatively isolated cortical effects of WFS. Cortical thickness was reduced in WFS in restricted regions, including lingual, precentral, and rostral middle frontal cortex. These cortical regions are associated with vision, motor function and higher order cortical functions such as working memory, respectively. These regions are known to reduce in thickness with age during middle childhood, and be influenced by genetic factors [79]. In addition, both increased [80] and decreased thickness can be seen in neurodegeneration and neurodevelopmental conditions [81]–[83]; however, mechanistic interpretations of this finding are unclear. Furthermore, other metrics, such as cortical area and volume did not reveal any significant effects, suggesting that cortical differences in early WFS are much more subtle and restricted than cerebellar and brainstem changes.

Our complementary, quantitative measurements of structural brain differences in WFS allow us to build hypotheses about potential underlying mechanisms. For example, we found reduced fractional anisotropy and reduced axial (but not radial) diffusivity in the brainstem, cerebellum, optic radiations and numerous other regions. Radial diffusivity, the diffusion of water perpendicular to white matter fibers, is known to increase in response to myelin damage [84]–[86]. In contrast, fractional anisotropy, the overall directionality of water movement, and axial diffusivity, the diffusion of water parallel to white matter fibers, is known to decrease in response to axonal damage [87]–[92]. Thus, it is reasonable to conclude that the differences observed in the WFS group’s white matter microstructure reflect either axonal damage or impaired axonogenesis. Although neuropathological case reports have also suggested loss of myelinated axons in these regions, it has not always been clear if these are degenerative or developmental changes [21], [76].

While WFS has previously been considered a neurodegenerative disease, it is also possible that the brain abnormalities reported in WFS are the product of two separate pathological processes, one that is neurodevelopmental in nature and the other neurodegenerative. Another possibility is that although there are early brain changes, these do not produce neurological symptoms until later in development, due to compensatory processes or the fact that the involved brain networks and functions do not come on-line until later [93]. For example, in our study and in others, the MRI and neuropathological changes observed appear more severe than the neurobehavioral findings would suggest [27], [30]. The apparent lack of cognitive deficits in our cohort in the context of highly atypical brain volumes (e.g. brainstem and cerebellum) could indicate compensatory processes or the lack of involvement of these regions in higher order cognitive processing. Notably, we found that many of these subjects have ataxia which has been associated with cerebellar dysfunction in other developmental populations [94]. Longitudinal follow-up of our cohort, and the addition of even younger and earlier, pre-symptomatic WFS patients will be essential for determining the difference between neurodevelopmental and neurodegenerative processes.

Our cohort also had notable depression and anxiety, similar to findings in advanced WFS [95] and anxiety behaviors found in animal models of WFS (wfs1-deficient mice) [96]. While cerebellar dysfunction has also been associated with altered mood [97], we cannot currently distinguish whether these symptoms are a reaction to living with a chronic, degenerative condition or if they are part of the disease process itself. Our findings suggest the need for psychiatric assessment and possibly treatment even in the early stages of WFS. Tracking changes in mood and anxiety will be an important component of future research.

The major strength of this study is its quantification of regional brain abnormalities in a group of WFS individuals with a range of disease severity, from newly diagnosed to more significantly affected. Although the WFS sample presented here is small compared to studies of more common neurodegenerative diseases, it is the largest quantified neuroimaging study of WFS to date. Furthermore, we compare our WFS to two larger groups of age and gender equivalent comparison groups, to control for any effects of diabetes or normal development on the brain, and we applied multiple comparison corrections. However, there are some limitations to our study. Since our study design was cross-sectional, we cannot be sure that the observed age-independence of certain effects would be confirmed in a longitudinal within-subjects design. Control subjects were a convenience sample obtained from several current studies at WUSM, and thus did not have cognitive/behavioral testing. Although all WFS participants who were willing to travel to St. Louis were accepted into the study, this requirement may have selected for individuals who were higher functioning than the general WFS population. The T1C subjects with DTI scans had shorter duration of diabetes than our WFS subjects. Ideally, groups would be matched on this variable and on degree of hyperglycemia and hypoglycemia exposure. Based on our experience and the existing literature, however, we feel that it is highly unlikely that small differences in duration of diabetes would cause the dramatic differences seen between WFS and the control groups (T1C and healthy). In all of the previous neuroimaging studies of T1C, including those in youth [98], [99] and in adults with much longer duration and extreme exposure to hyperglycemia and hypoglycemia [100], [101], none have found the pattern of dramatically reduced brainstem and cerebellar volumes seen here in the WFS. Groups also had slight differences in the sequences and scanners used, although these relatively minor differences would not be expected to explain the dramatic findings in our WFS group. Furthermore, when the data analyses were restricted to only those subjects scanned on the same machine using identical sequences, our findings remained highly significant.

The results of this study lay the groundwork for larger, longitudinal investigations of neuroanatomical changes in WFS which could confirm and extend our observations, and distinguish between earlier vs. later occurring abnormalities. In addition, the behavioral correlates of neuroanatomical changes will need to be better understood. For example, functions known to be reliant on the optimal development of the cerebellum and brainstem, such as gait and balance, should be better characterized. Longitudinal measurement of this cohort of individuals with WFS is ongoing and will need to be significantly expanded to answer these important clinical questions. Nevertheless, our current data provide fundamental insight into the neurophenotype associated with WFS and provide the necessary information for determining the appropriateness of animal models of WFS for understanding the neurobiological mechanisms underlying these findings and for developing neurologically-targeted interventions. This work would also contribute to our understanding of the impact of ER stress-related dysfunction during development.
